# Barriers to uptake of referral services from secondary eye care to tertiary eye care and its associated determinants in L V Prasad Eye Institute network in Southern India: A cross-sectional study-Report II

**DOI:** 10.1371/journal.pone.0303401

**Published:** 2024-05-14

**Authors:** Debananda Padhy, Giridhar Pyda, Srinivas Marmamula, Rohit C. Khanna

**Affiliations:** 1 Allen Foster Community Eye Health Research Centre, Gullapalli Pratibha Rao International Centre for Advancement of Rural Eye Care, L V Prasad Eye Institute, Hyderabad, India; 2 Brien Holden Eye Research Centre, L V Prasad Eye Institute, Hyderabad, Telangana, India; 3 School of Medicine and Dentistry, University of Rochester, Rochester, NY, United States of America; 4 School of Optometry and Vision Science, University of New South Wales, Sydney, Australia; Irrua Specialist Teaching Hospital, NIGERIA

## Abstract

**Aim:**

To investigate the barriers to the uptake of referral services from secondary care centers (SC) to a higher-level tertiary care center (TC) in Southern India.

**Methods:**

A cross-sectional study was conducted in the Mahabubnagar district of Telangana, India, between February 1, 2018 to January 31, 2019 and all those referred from SC to TC between January 1, 2013 to December 30, 2016 were identified for interview. Based on inclusion criteria, of the 960 participants identified, 681 (70.9%) participated in the study. A validated study questionnaire was administered to all participants. Information collected were the demographic details, details related to their referral and barriers to referral. The participants that presented at TC were considered compliant and who did not, were non-compliant. Reasons for non-compliance was also collected.

**Results:**

The mean age those interviewed was 46.1 years (SD: 17.3 years) and 429 (63%) were males and 252 (37%) were females. Overall, 516 (75.8%) were compliant, and 165 (24.2%) were non-compliant. The major factors for non-compliance were economic (16.4%) and attitudinal (44.2%) barriers. Within the attitudinal barrier category, the most prevalent individual attitudinal barriers were ‘too busy to go to the eye center for treatment (16.4%)’and ‘able to manage routine daily activities with current vision (12.1%)’. The multivariable analysis showed that the non-compliant participants had only visited the SC once prior to the referral (odds ratio: 2.82; 95% CI: 1.43–5.57) (p = 0.003).

**Conclusions:**

Participants with only one SC visit, were less likely to comply with referrals and the major barriers to compliance were economical and attitudinal. It is important to address these specific barriers to provide proper counseling to participants during referrals.

## Introduction

Globally 2.2 billion people have vision impairment (VI) of which one billion have VI which is either preventable or yet to be addressed [1]. The major barriers to the use eye care services were related to availability, accessibility and acceptability of services, especially in developing countries [1]. In developing countries, the availability of eyecare services is frequently restricted, especially in rural areas [1]. Even when they are available, there are many barriers inhibiting access to these services [2]. The most significant obstacles to the uptake of eyecare are the cost [3], fear of surgery [4], unavailability of personal support or transportation [5, 6], paucity of a perceived necessity [3], and poor surgical outcomes in others [7]. A number of other factors, including age and sex, education levels, marital status, socioeconomic status, healthcare cost, type and severity of illness, distance and physical access and perceived quality of services may also act as obstacles to the uptake of eye care services [8].

The World Report on vision (WRV) has proposed an integrated care model, including primary, secondary, and tertiary care, with clear delineation and demarcation of function and appropriate referrals at each level of care. We have a similar model in our institute in India [9]. Compliance with referrals is necessary to ensure the completion of the treatment cycle. However, not all referred patients present at the higher tier centers [7]. There are numerous studies on barriers to access to eye care services, however there are hardly any study looking at the barriers to access to care when someone is referred to next level of care. Understanding of these barriers would help us in planning strategies to improve access to care for these referral patients. So far, only one study has been conducted which examines the barriers to referral services to higher centres [10]. This is the second study of a similar kind which reports the obstacles to the uptake of referrals to higher-level services, and the associated risk factors in the Mahabubnagar district of Telangana, India.

## Methods

This study was approved by the institutional ethics committee of the institute. The study adhered to the tenets of the Declaration of Helsinki. This is a cross-sectional study carried out at a secondary center (SC) of the Institute, namely Kuchakulla Ramachandra Reddy Eye Centre (KRREC), Thoodukurthy village, Mahabubnagar district (erstwhile), Telangana, South India. Overall, the literacy rate for Mahabubnagar district is 55.04% (males– 65.2% and females– 44.7%) and agriculture is the main occupation. We reviewed the electronic medical records of patients referred to the tertiary center (TC) from KRREC between January 1, 2013 to December 30, 2016 and the study was conducted between February 1, 2018 and January 31, 2019. The inclusion criteria were participants aged more than or equal to 18 years, living within a 50-kilometer radius of KRREC, referred to TC from SC, having phone contact information, and being willing to participate. Participants were considered as compliant if they attended a TC after being referred and non-compliant if they failed to follow up at TC within one year of referral.

A validated study questionnaire that was administered in our previous study was re-used in this study by three trained field investigators [10]. Written informed consent was obtained from all participants prior to the administration of the questionnaire. The study methodology was similar to that described in our previous related publication [10]. In brief, the trained investigators contacted the participants initially via telephone one week prior to visit and those contacted were visited in-person. While visiting them, enquiry was also made about other participants who could not be contacted telephonically and if available, they too were visited in-person to their given addresses. The following information was obtained from the medical records: demographic details, the number of visits to the eye center prior to referral; the participant’s unaided, presenting, and aided visual acuity at the time of referral; the purpose for the referral; the department that the participant was sent to, such as the cornea, oculoplastics, neuro-ophthalmology, glaucoma, retina, low vision, rehabilitation, and other referral. The status was categorized as paying or non-paying. The classification is based on a tiered system of patient care, catering to both paying and non-paying patients. Paying category consists of patients who pay the standard rates for consultations, surgeries, and other services. Conversely, non-paying services are offered entirely free of charge to economically underprivileged individuals, encompassing comprehensive eye care, surgeries, and rehabilitation services. Trained field workers went door-to-door to administer the referral uptake questionnaire. A list of barriers was included in the questionnaire, which was categorized based on economics, knowledge, logistics, and attitudes and a single most barrier was selected as the main barrier for the study. The compliant participants were asked why they chose to receive additional care at the TC. The non-compliant participants were asked to mention the main reasons for non-compliance. Participants were considered not available if they were not at home after three visits from the field staff spaced at least a week apart.

### Statistical analysis

Following data collection, all the data forms were checked for completion and accuracy. They were brought to the data center located in Hyderabad, and the consistency check was done. Double entries were made to minimize data entry errors. The WHO categories of visual impairment were used for analysis, which categorized vision based on presenting visual acuity (VA) in the better eye (i.e., normal for VA of 6/18 or better, moderate visual impairment for less than 6/18 to 6/60, severe visual impairment for less than 6/60 to 3/60, and blind for anything lower than 3/60) [11]. The Student’s t-test was used for analyzing the continuous variables. The chi-square test or Fisher’s exact test was used as appropriate for the categorical variables. Univariable and multivariable logistic regression analyses were performed to examine the risk factors for non-compliance. The Hosmer-Lemeshow goodness of fit model was used to evaluate the fit of the logistic regression model. A two-sided p-value <0.05 was considered statistically significant. All the data were entered using Microsoft access software and analyses were performed using Stata 16.1 version software (College Station, TX: StataCorp LLC) for windows.

## Results

Between January 2013 and December 2016, 960 participants were referred to the TC in Hyderabad. Among the 960, 681 (70.9%) were interviewed. Of these participants, 516 (75.8%) were compliant, and 165 (24.2%) were non-compliant. The remaining 279 (29.1%) participants were unavailable. The reasons for their unavailability are as follows: 52 (5.4%) died, 31 (3.2%) changed their address, 43 (4.5%) provided wrong contact numbers, 28 (2.9%) did not respond to the call, and 125 (13.0%) were not traceable.

The mean age was 46.1 years (SD: 17.3 years) in those interviewed and 49.1 years (SD: 18.5 years) in those not interviewed; the difference was statistically significant (p = 0.020). Among those interviewed 429 (63%) were male and 252 (37%) were female. There was no significant difference between interviewed and non-interviewed groups for gender (male: female; 72.6%:68.3% versus 27.4%:31.7%; p = 0.165) and economic status (paying: non-paying; 71.1%:70.1% versus 28.9%:29.9%; p = 0.784). Table 1 shows the differences in the demographic, socioeconomic, and ocular factors of the compliant and non-compliant groups. The non-compliant participants were more likely to be non-paying (p = 0.011), had visited the SC only once (p = 0.004), were mostly illiterate (p = 0.039), had VI (p = 0.056) and were widowed or separated(p = 0.059).

**Table 1:** Differences in demographic, socioeconomic, and ocular factors between the compliant and non-compliant groups.

Factors	Non-compliant(N = 165)	Compliant (N = 516)	P-value
	n (percentage)	n (percentage)	
Age group (years)(Median)			
18–45 (< = 45 years)	84(50.9)	255(49.4)	0.739
46–93 (>45 years)	81(49.1)	261(50.6)	
Gender			
Male	94(57.0)	335(65.0)	0.066
Female	71(43.0)	181(35.0)	
Status			
Paying	124(75.2)	433(83.9)	**0.011**
Non-paying	41(24.8)	83(16.1)	
Mean distance from Secondary centres (kilometre)	36.5(19.8)	38.2(21.7)	0.338
Visit to secondary centre			
More than one visit	147(89.1)	492(95.3)	**0.004**
One visit	18(10.9)	24(4.7)	
Presenting visual acuity (PVA) in better eye			
Normal (better than or equal to 6/18)	100(60.6)	356(68.9)	**0.046**
Any Visual impairment (worse than 6/18)	65(39.3)	160(31.0)	
Referral clinic			
Paediatric & Neuro-ophthalmology	12(7.3)	36(7.0)	0.418
Cornea	42(25.5)	124(24.0)	
Glaucoma	9(5.5)	25(4.8)	
Retina	37(22.4)	161(31.2)	
Low vision	5(3.0)	14(2.7)	
Oculoplasty	37(22.4)	85(16.5)	
Others	23(13.9)	71(13.8)	
Marital status			
Married	116(70.3)	404(78.3)	**0.059**
Unmarried	25(15.2)	67 (13.0)	
Widowed & separated	24(14.5)	45(8.7)	
Education			
No education	99(60.0)	262(50.8)	**0.039**
Formal education	66(40.0)	254(49.2)	
Earning member			
Yes	61(37.0)	236(45.8)	0.140
No	47(28.5)	129(25.0)	
Contribute to family income	57(34.5)	151 (29.2)	

Table 2 shows the univariable and multivariable analyses for factors for non-compliance. The univariable analysis showed that non-compliance is significantly associated with non-paying, having only one visit to the SC, having a visual impairment, being widowed or separated, and being illiterate. The multivariable analysis showed that non-compliance was significantly associated with those who visited SC only once (adjusted Odds Ratio (OR): 2.82 (95% CI: 1.43–5.57).

**Table 2:** Univariable and multivariable analyses of the risk factors for non-compliance.

Factors	Compliant (n = 516)		Non-Compliant (n = 165)	
	Univariable OR (95% CI)	P-value	Multivariable OR (95% CI)	P-value
Age group(years)(Median)				
18–45 (< = 45 years)	1.00		1.00	
46–93 (>45 years)	0.94(0.66–1.34)	0.739	0.80(0.519–1.24)	0.330
Gender				
Male	1.00		1.00	
Female	1.40(0.98–1.99)	0.066	1.00(0.65–1.53)	0.980
Status				
Paying	1.00		1.00	
Non-paying	**1.73(1.13–2.64)**	**0.012**	1.54(0.94–2.53)	0.086
Mean distance from Secondary centres (kilometre)	1.00(0.99–1.00)	0.338	0.99(0.98–1.00)	0.135
Visit to secondary centre				
More than one visit	1.00		1.00	
One visit	**2.51(1.33–4.75)**	**0.005**	**2.82(1.43–5.57)**	**0.003**
Presenting visual acuity (PVA) in better eye				
Normal (better than or equal to 6/18)	1.00		1.00	
Any Visual impairment (worse than 6/18)	**1.44(1.00–2.08)**	**0.047**	1.48(0.97–2.26)	0.066
Referral clinic				
Paediatric & Neuro-ophthalmology	1.00		1.00	
Cornea	1.02(0.48–2.13)	0.966	0.98(0.45–2.11)	0.960
Glaucoma	1.08(0.40–2.93)	0.881	1.00(0.35–2.86)	0.988
Retina	0.69(0.33–1.45)	0.328	0.70(0.32–1.52)	0.373
Low vision	1.07(0.32–3.60)	0.911	0.91(0.25–3.25)	0.889
Oculoplasty	1.31(0.61–2.79)	0.491	1.34(0.60–2.95)	0.468
Others	0.97(0.44–2.17)	0.945	0.73(0.31–1.71)	0.470
Marital status				
Married	1.00		1.00	
Unmarried	1.30(0.79–2.15)	0.308	1.17(0.62–2.19)	0.620
Widowed & separated	**1.86(1.09–3.18)**	**0.024**	1.63(0.88–3.00)	0.114
Education				
Formal education	1.00		1.00	
No education	**1.45(1.02–2.08)**	**0.039**	1.37(0.87–2.14)	0.165
Earning member				
Yes	1.00		1.00	
Contribute to family income	1.46(0.97–2.21)	0.074	1.42(0.89–2.25)	0.132
No	1.41(0.91–2.18)	0.123	1.41(0.86–2.32)	0.168

95% CI: 95% Confidence Interval; OR: adjusted Odds Ratio

Table 3 shows the major barriers to the uptake of referrals reported by the non-compliant group. The most common barriers reported were economical (16.3%) and attitudinal (44.2%). Within the attitudinal barrier category, the most prevalent individual attitudinal barriers were ‘too busy to go to the eye center for treatment (16.4%)’and ‘able to manage routine daily activities with current vision (12.1%)’.

**Table 3:** The major barriers for the uptake of referrals in the non-compliant group.

Categories	Major Barriers	Numbers (%)
Economics	Cannot afford to travel cost to the tertiary center	11(6.7)
	Cannot afford treatment costs	11(6.7)
	Cannot afford lost wages of me or accompanying person	5(3.0)
Knowledge	Do not know where the tertiary center in Hyderabad	4(2.4)
	Do not understand the need to go to tertiary center	2(1.2)
	Heard that vision would not improve	16(9.7)
	Not aware of referral	4(2.4)
Logistics	Nobody to accompany to tertiary center	8(4.8)
	The tertiary center is very far from home	7(4.2)
	Other health problems prevent from travelling to eye center	13(7.9)
	L V Prasad did not help facilitate treatment	4(2.4)
Attitudes	Fear of travelling to tertiary center	5(3.0)
	Fear of procedure	3(1.8)
	Too busy to go to eye center for treatment	27(16.4)
	Dominant family member does not feel need	6(3.6)
	Able to see adequately i.e. ’able to manage routine daily activities with current vision’	20(12.1)
	Old age-do not see the need for treatment at my age	5(3.0)
	Not satisfied with the treatment at SC	4(2.4)
	Decided to visit another eye center	3(1.8)
Others	Other	7(4.2)

## Discussion

This is the second study we conducted that looks at the non-compliance for the uptake of referral services from SC to TC in our network. Data from our study shows that nearly 25% of referrals were non-compliant. ‘Too busy to go to the eye center for treatment’ and ‘Able to manage routine daily activities with current vision’ were the major reasons for non-compliance. The Mahabubnagar district mostly comprises daily wage workers. It is possible that these participants did not want to lose their daily wages and deferred the uptake of referral services. They might have also felt that their needs were low, as they could manage their daily tasks with their current vision. As major barriers were related to attitudinal, there is a need to develop a behavioral change strategy with more in-depth focus group discussions and in-depth interviews. In our study, 16.4% of participants identified financial constraints as an impediment to accessing referral services. This finding aligns with previous literature demonstrating the economic challenges faced by participants with vision impairment in developing countries [12]. Participants often face challenges in affording expenses related to transportation, treatments, and taking time off from work to attend medical services, driven by concerns about their daily income [13]. Addressing these financial barriers through targeted interventions will be crucial for improving access to services for this vulnerable population.

A factor for the poor uptake of referral services is those with only one visit to the SC prior to referral. This could be because of the limited understanding of the healthcare system in our network. Similar results were seen in our previous study [10]. Therefore, adequate counseling about the referral system and health literacy is required for each referral. In developing nations, most people from low-income households lack basic literacy and knowledge about eye health [14]. Previous studies have reported underuse of eyecare services exacerbated by a wide range of public health problems, such as gender inequality [15], illiteracy, unemployment [16], low socioeconomic level [17], and cost [18]. Some low-income communities believe that receiving eye care may deteriorate their vision [19]. The lack of awareness keeps people from using these services, and the harmful conditions go unchecked and unmanaged. Apart from this, fear of testing and a lack of adequate knowledge about serious eye conditions could be detrimental for not seeking care. Therefore, it is crucial to be aware of the options available in eye health, treatment alternatives, and myths. Hence Appropriate health literacy and counseling will help improve the uptake of referral services by non-paying participants. A recently adopted strategy was a specialist from TC traveling to the SC to provide specialty care at the doorstep. This strategy might improve the uptake of services, especially by the non-paying participants. Apart from this, another strategy which classifies referrals as ‘Emergency’ and ‘Non-Emergency,’ and a system to track emergency referrals would ensure the uptake of referral services.

A strength of this study is being carried out in an eye health system, which provides information on the poor uptake of referral services in a network of eye hospitals in India. It also identifies risk factors for poor compliance for the uptake of services so that strategies can be designed to negate them. This study has some limitations. The research was done in a particular healthcare system in South India. Therefore, its conclusions cannot be extrapolated and generalized. Another limitation is the cross-sectional design of the study, which emphasizes associations over direct causality. Apart from this, we did not use the theoretical domain framework (TDF)models which uses Focus Group Discussions (FGDs) and in-depth interviews which is a better way to understand the barriers and provide best intervention strategies to overcome the barriers. Some of the factors which can also affect compliance like hospital waiting time, accessibility of the hospital, and attitude of the staff etc. were not collected.

## Conclusions

In conclusion, approximately 25% were non-compliant with referral uptake from SC to TC. We have found that people with only one SC visit are non-compliant with referral services from SC to TC. Major barriers to the uptake of referrals are related to attitude. Therefore, there is a need for appropriate health education and awareness creation among this population. This population should be made aware that most vision problems are curable, and VI can be avoided by utilizing the suggested eye care services.
